# Insulin Treatment May Alter Fatty Acid Carriers in Placentas from Gestational Diabetes Subjects

**DOI:** 10.3390/ijms18061203

**Published:** 2017-06-06

**Authors:** Maria Ruiz-Palacios, Maria Teresa Prieto-Sánchez, Antonio José Ruiz-Alcaraz, José Eliseo Blanco-Carnero, Maria Sanchez-Campillo, Juan José Parrilla, Elvira Larqué

**Affiliations:** 1 Department of Physiology, Faculty of Biology, Campus Mare Nostrum, University of Murcia, Murcia 30100, Spain; maria_coina_200@hotmail.com (M.R.-P.); medit2011@gmail.com (M.S.-C.); 2 Obstetrics and Gynecology Service, Virgen de la Arrixaca Clinical Hospital, University of Murcia, Murcia 30120, Spain; mariatemedicina@hotmail.com (M.T.P.-S.); jeblancoc@gmail.com (J.E.B.-C.); jjparri@um.es (J.J.P.); 3 Department of Biochemistry, Molecular Biology B and Immunology, Campus Mare Nostrum, University of Murcia, Murcia 30100, Spain; ajruiz@um.es

**Keywords:** gestational diabetes, placental lipid transport, insulin resistance, fetal adiposity

## Abstract

There is little information available on the effect of Gestational diabetes mellitus (GDM) treatment (diet or insulin) on placental lipid carriers, which may influence fetal fat accretion. Insulin may activate placental insulin receptors protein kinase (AKT) and extracellular signal regulated kinase ERK mediators, which might affect lipid metabolism. Placenta was collected from 25 control women, 23 GDM-Diet and 20 GDM-Insulin. Western blotting of insulin signaling mediators and lipid carriers was performed. The human choricarcinoma-derived cell line BeWo was preincubated with insulin inhibitors protein kinase (AKT) and extracellular signal regulated kinase (ERK) and ERK inhibitors to evaluate insulin regulation of lipid carriers. Maternal serum insulin at recruitment correlated to ultrasound fetal abdominal circumference in offspring of GDM and placental endothelial lipase (EL). Lipoprotein lipase in placenta was significantly reduced in both GDM, while most of the other lipid carriers tended to higher values, although not significantly. There was a significant increase in both phosphorylated-Akt and ERK in placentas from GDM-Insulin patients; both were associated to placental fatty acid translocase (FAT), fatty acid binding protein (A-FABP), and EL. BeWo cells treated with insulin pathway inhibitors significantly reduced A-FABP, fatty acid transport protein (FATP-1), and EL levels, confirming the role of insulin on these carriers. We conclude that insulin promotes the phosphorylation of placental insulin mediators contributing to higher levels of some specific fatty acid carriers in the placenta and fetal adiposity in GDM.

## 1. Introduction

The mechanisms that enhance fetal fat accretion in well glucose-controlled Gestational diabetes mellitus GDM pregnancies are largely unknown. GDM alters the placental structure which may affect nutrient transport to the fetus [1]. Fatty acid uptake by the placenta requires the activity of several lipases including lipoprotein lipase (LPL) and endothelial lipase (EL), which produce non-esterified fatty acids (NEFA) [2,3]. NEFA can enter the placenta by simple diffusion or by using fatty acid carriers like fatty acid translocase (FAT/CD36) [4], plasma membrane fatty acid-binding protein (FABPpm) [5] or fatty acid transport proteins (FATPs) [6]. Once fatty acids are in the cytosol, they bind to fatty acid binding proteins (FABP) [7] before being transferred to the fetal circulation and for intracellular trafficking. Several studies have investigated the effect of GDM on lipid carriers in the placenta, but using a low number of subjects and without considering the mode of GDM treatment (diet or insulin) [8,9], which may influence lipid transport. 

Insulin signaling is critical for the regulation of both intracellular and blood glucose in GDM. Insulin cannot cross the placenta [10], but it can bind to its specific receptor IR (insulin receptor) in the trophoblast membrane [11] to activate insulin signaling pathways via Ras-ERK (extracellular-signal-regulated kinase) and the IRS (insulin receptor substrate)-PI3kinase-Akt-mTOR (mammalian target of rapamycin) [12,13]. Colomiere et al. suggested the existence of post-receptor defects in the insulin signaling pathway in placentas from GDM treated with diet vs. insulin, although the limited number of subjects of that study (less than 10) could limit relevance of the results [14]. Limited data is available on the level of activated insulin mediators in the placenta of GDM and their consequences on placental-fetal metabolism.

The clinical GDM treatment might affect both maternal metabolism and the expression of lipid carriers in the placenta, and it might enhance fetal fat accretion. Both FAT and FATP-1 seem to be modulated by insulin mediators such as Akt in peripheral tissues like heart [15], adipocytes, and skeletal muscle [16], while little data is available on placental tissue. Only placental LPL modulation by insulin has been reported in humans [17], but no information is available for the rest of placental fatty acid carriers. The aim of this study was to investigate the effect of insulin in both placental lipid carriers and insulin mediators in GDM women following different clinical treatments (diet or insulin). We also confirmed the effect of insulin on lipid carriers by in vitro experiments using the human placenta choricarcinoma-derived BeWo cell line.

## 2. Results

### 2.1. Subject Characteristics

The fetal abdominal circumference *z*-score (z-AC), tended to be higher in GDM (*p* = 0.071) pointing to higher fat accretion in these babies. In fact, these differences were statistically significant when the GDM-Insulin was directly compared with the controls (*p* = 0.02) by student *t*-test. GDM mothers had significantly higher BMI than controls before pregnancy, in the 3rd trimester and at delivery (Table 1).

Placental thickness and weight were higher in both GDM groups, which might affect placental fatty acid transport (Table 1). Maternal glucose and insulin were significantly higher in GDM at the third trimester before any treatment (recruitment); at delivery, only maternal glucose remained significantly higher in the GDM, although still within the normal clinical range, while insulin tended to higher levels in the GDM-Insulin (*p* = 0.067) (Table 1). Maternal insulin at recruitment correlated to both z-AC at recruitment (*r* = 0.266, *p* = 0.025) and at delivery (*r* = 0.275, *p* = 0.023). Maternal TG at recruitment was also significantly higher in the GDM-Insulin with the same trend at delivery. Z-AC tended also to be associated to TG at recruitment (*r* = 0.207, *p* = 0.079). TG and total fatty acids in cord blood were both significantly lower in GDM, in line with enhanced fetal adipose storage (Table 1).

### 2.2. Lipases and Lipid Carriers in Placentas from GDM

**Figure 1:**
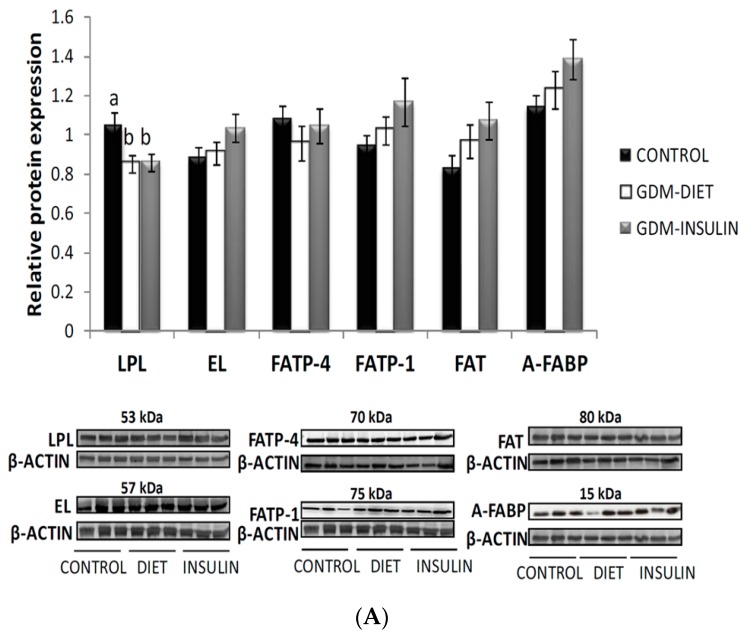

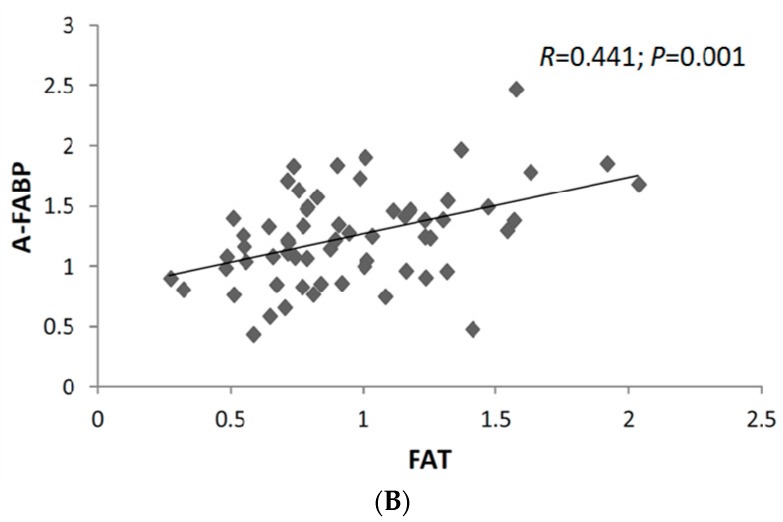
(**A**) Relative protein expression normalized to β-Actin of placental lipases, lipoprotein lipase (LPL) (*p* = 0.030) and endothelial lipase (EL), and lipid carriers fatty acid binding protein (A-FABP), fatty acid translocase (FAT), fatty acid transport protein (FATP-1) and fatty acid transport protein (FATP-4) in placental tissue from control and gestational diabetes mellitus (GDM) patients. Results are expressed as Mean ± SEM). ANOVA followed by a Bonferroni test was used to assess differences among the groups. Different letters over the bars indicate significant differences (*p* < 0.05); (**B**) Correlation between placental FAT and A-FABP protein expression.

Contradictory results on placental lipases were found. LPL was significantly reduced in GDM (*p* = 0.030), while most of the other carriers tended to higher values, although the differences were not significant (Figure 1A). Membrane placental protein FAT correlated significantly with cytosolic A-FABP (Figure 1B), which might enhance fat storage within placental lipid droplet structures.

### 2.3. Phosphorylated Insulin Signaling in GDM Placentas

**Figure 2:**
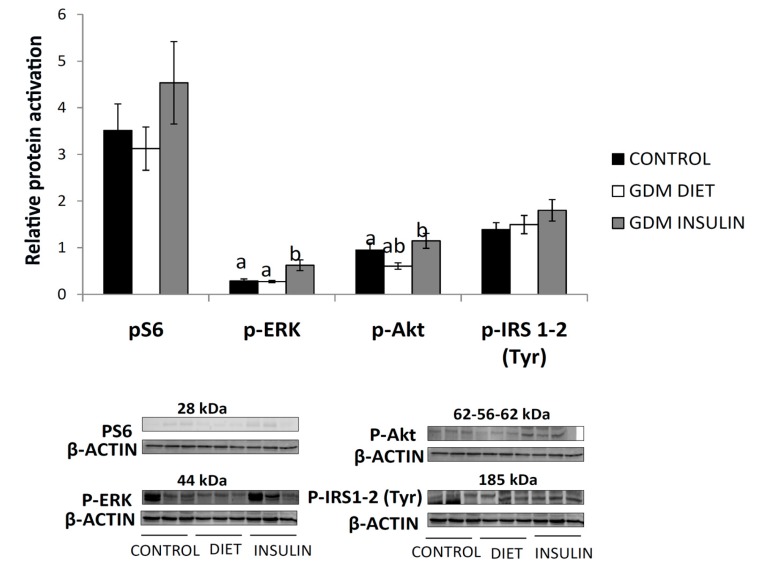
Relative protein activation normalized to β-Actin of phosphorylated insulin signaling mediators, Ribosomal protein S6 (pS6), phosphorylated extracellular signal regulated kinase (p-ERK), phosphorylated protein kinase B (p-Akt) and phosphorylated insulin receptor substrate-1 (p-IRS1-2) (Tyr) in placentas from control and gestational diabetes mellitus (GDM) patients. Results are expressed as (Mean ± SEM). ANOVA followed by a Bonferroni test was used to assess differences among the groups. Different letters over the bars indicate significant differences (*p* < 0.05).

Both, phosphorylated Akt and ERK increased significantly in placentas from the GDM-Insulin (Figure 2). p-Akt signaling tended to be reduced in the GDM-diet group, and in fact, it was significantly different if compared directly between the Control and GDM-diet by *t*-test, which might indicate a certain degree of insulin resistance.

**Figure 3:**
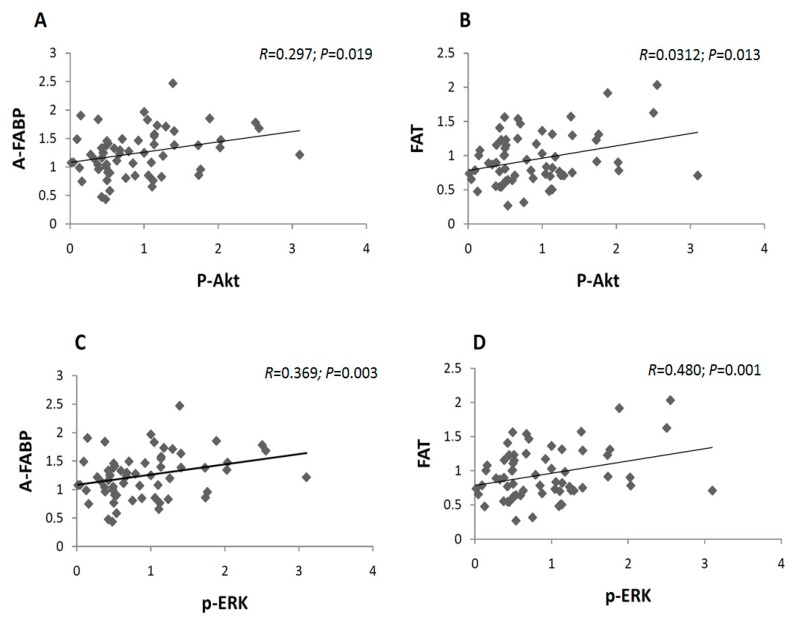
Correlations between fatty acid carriers and phosphorylated insulin signaling mediators in placentas, from control and GDM groups. (**A**) Correlation of fatty acid binding protein (A-FABP) with phosphorylated protein kinase B (p-Akt); (**B**) Fatty acid translocase (FAT) with p-Akt; (**C**) A-FABP with phosphorylated extracellular signal regulated kinase (p-ERK); (**D**) FAT with p-ERK.

Phosphor-S6 (p-S6) was not statistically significant due to high variability in its results. Both Akt and ERK were correlated with both placental FAT and A-FABP (Figure 3), suggesting that the insulin signaling pathway could be involved in fat accretion in GDM babies. Moreover, EL was also associated to p-AKT (*r* = 0.374, *p* = 0.003) and to maternal insulin at recruitment (*p* = 0.325, *p* = 0.014).

### 2.4. In Vitro Effect of Insulin on Lipid Carriers in BeWo Cells

**Figure 4:**
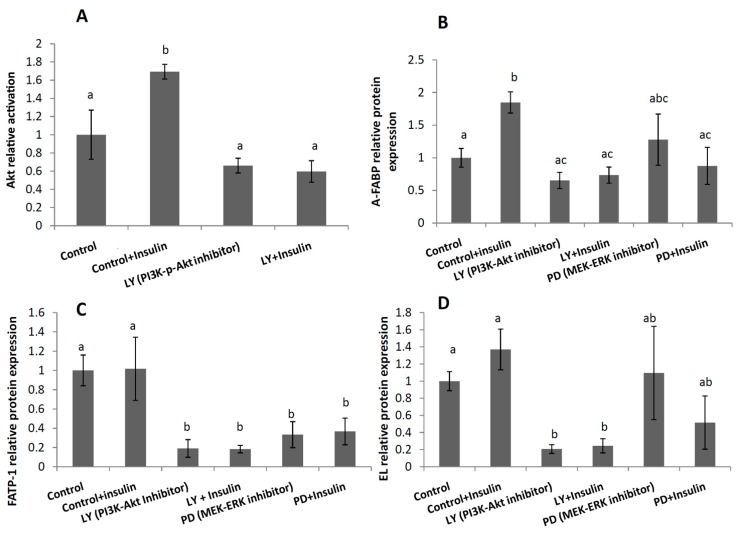
Relative protein activation/expression of protein kinase B Akt/fatty acid carriers normalized to β-Actin of: (**A**) p-Akt, (**B**) Fatty acid binding protein A-FABP (**C**) Fatty acid transport protein FATP-1 and (**D**) Endothelial lipase EL in BeWo cells preincubated 1 h with PI3K-Akt (LY294002) and MEK-ERK (PD98059) pathway inhibitors (50 μM) and stimulated with insulin (10 nmol/L) for 24 h. Results are expressed as Mean ± SEM. A student *t*-test was used to assess differences among the groups. Different letters on the bars indicate significant differences (*p* < 0.05).

As expected, phosphor-Akt was significantly higher in insulin stimulated BeWo cells compared with controls (Figure 4A).

A-FABP increased significantly in the insulin treated cells, whereas only a slight trend was observed for EL and FATP-1 (Figure 4B–D). To ascertain whether insulin could be responsible for higher fatty acid carriers in GDM placentas, we also preincubated BeWo cells with Akt and ERK inhibitors (PI3K and MEK, respectively) (Figure 4). Both A-FABP and EL were reduced by the PI3K inhibitor, but not by the MEK inhibitor, demonstrating the important role of PI3K-Akt in the modulation of both A-FABP and EL. FATP-1 was reduced by both the PI3K-Akt and the MEK-ERK inhibitors, confirming the role of these two pathways in the activation of this carrier by insulin (Figure 4).

## 3. Discussion

The effects of different GDM clinical treatments (diet or insulin) on the activation of downstream insulin signaling mediators in human placenta were studied, finding slight placental insulin resistance in the GDM-Diet, while such resistance was overcome by insulin treatment. In addition, using both in vivo and in vitro studies, we demonstrated for the first time the relationship between insulin pathway mediators and enhanced fatty acid carriers in human placenta, which could be one of the mechanisms involved in fetal fat accretion in GDM.

Systemic measurements of glucose status suggest that the insulin-treated women were hyperglycemic despite hyperinsulinemia, which may affect birth weight, and the HAPO study also reported a strong correlation between increased maternal glucose and fetal adiposity, even when glucose values were below the pathological limits [18,19]. Fetal z-AC, which is a prenatal parameter of fetal adiposity [20], was associated to insulin and TG at the beginning of third trimester, suggesting that maternal insulin is affecting materno-fetal structures from that time. An earlier intervention in GDM patients could be appropriate to reduce fetal adiposity. Moreover, newborns from GDM mothers treated with insulin tended to the highest values of AC, which could be explained by the enhanced lipid carriers in placentas and higher storage of fat in adipose tissue in the fetus, although other mechanisms could also occur. Both the decrease in serum TG and total fatty acids in cord blood could explain higher fetal fat storage, as suggested by Schaefer-Graf et al. [21]. A previous study suggests that the placenta could be mainly responsive to maternal insulin in early pregnancy, but not during late pregnancy [22]. This may explain why maternal insulin at recruitment correlated with z-AC.

Currently, to understand the role of insulin on GDM pathophysiology and fetal macrosomia is a challenge. Since insulin cannot cross the placental tissue, its effect on placental metabolism has been underestimated. Nevertheless, insulin plays a major role in the regulation of the placental nutrient metabolism; up-regulation of some placental amino acid transporters, through activation of mammalian target of rapamycin (mTOR) signaling by insulin and IGF-1, was reported in obese women giving birth to large babies [23]; similar results were found in cultured trophoblast cells [24]. Here, we demonstrate that insulin clinical treatment might activate lipid carriers through the mediation of Akt and ERK in GDM patients. Thus, a significant increase of both phosphorylated Akt and ERK was confirmed in the GDM-Insulin. Our results agree with those of Colomiére et al. who found a significant decrease in the regulatory p85α subunit of PI3kinase in placentas of a GDM-Insulin, but not in the GDM-Diet; they suggested activation of the Akt signal in the GDM-Insulin, although they did not measure such intermediates [14].

The Ras-ERK insulin pathway is another cascade of insulin receptor activation, which has been less studied in insulin resistance. ERK mediates cell proliferation effects, whereas IRS-PI3K-Akt has been mainly implicated in metabolic actions of insulin [13]. In placental tissue there are no data available for the Ras-ERK pathway in GDM. Here, we demonstrate, for the first time, significantly higher phosphorylated ERK in placental tissue of GDM-Insulin patients, while no changes were observed between the control and GDM-Diet groups. Recent study has demonstrated defects in ERK signaling that could be responsible for insulin resistance in the skeletal muscle of women with polycystic ovary syndrome, but found no changes in Akt signaling [25]. Moreover, in skeletal muscle of obese subjects alterations in ERK activation have also been demonstrated [26]. The meaning and consequences of the alteration of ERK pathway in placenta merit further study, since this molecule seems to be a key for the proper response of placenta to insulin stimulation.

In the present study, fatty acid carriers A-FABP, FAT and EL gradually increased in the GDM-Diet and GDM-Insulin, but the differences between groups were not statistically significant. However, there was a significant reduction in placental LPL in both GDM, probably to counteract excessive fat accretion in the babies, as reported by Radaelli et al. [9]. Hyperglycemia reduces fatty acid oxidation and increases triglyceride accumulation in GDM [27], which may contribute to fatty acid delivery to the fetus. Both p-ERK and p-Akt correlated with the lipid carriers FAT and A-FABP, but also to EL, underlining the insulin-fat storage relationship within the placenta. Moreover, other factors apart from insulin signaling might also contribute to the trend to enhance fatty acid carriers in the GDM-Diet.

In order to ascertain whether fatty acid carriers depend on insulin receptor activation, we treated BeWo cells with insulin, and with or without Akt and ERK inhibitors (PI3K and MEK, respectively). First, we demonstrated a positive Akt response to insulin treatment by the increase in p-Akt, thus validating BeWo cells as a suitable model to test insulin influence on metabolism. Secondly, we showed that PI3K inhibition reduced A-FABP, EL, and FATP-1 proteins, indicating that Akt phosphorylation plays a major role in the regulation of placental lipid carrier. FAT protein was not detectable in these cells, thus we could not corroborate the association reported on maternal serum insulin and placental FAT. Nevertheless, Chabowski et al. already reported the translocation of FAT from the cytosol to the plasma membrane via Akt activation in cardiac myocites, while this effect was not observed for FABPpm [28]. Our results are also in agreement with Wo et al., who demonstrated the insulin modulation of FATP-1 via Akt in adipocytes and skeletal muscle [16]. Thus, insulin treatment in the GDM-insulin induced Akt and lipid carrier activation in the placenta.

We demonstrated the in vitro insulin regulation of EL, FATP-1, and A-FABP in placenta. In clinical trials, EL was high in placentas from the type I diabetes patients, who obviously required insulin [29]. Gauster et al. reported higher EL in placentas from the obese GDM than controls, but this effect did not occur in lean GDM, although they did not indicate whether the GDM obese group received insulin treatment [30]. Similarly, A-FABP was reported to be high in placentas from the obese-GDM, which could be due to the insulin treatment received by these subjects [31]. In fact, A-FABP was elevated in trophoblast cells when stimulated with insulin plus fatty acids, but not when stimulated with insulin alone [31]. In the present study, A-FABP was the most sensitive carrier to insulin stimulation at 24 h from all those we analyzed. As a limitation of the present study, to indicate that this time period of stimulation was used according to the bibliography to evaluate lipid metabolism response in cells [17,31]. We cannot discard that other times of insulin incubation might differ on the fatty acid carrier response. In addition, higher number of patients in the in vivo study could confirm the trend of some results observed on the lipid carriers. Nevertheless, these trends were supported with the experiments of the in vitro study.

In conclusion, hyperinsulinemia and hyperdislipidemia at the beginning of the third trimester disturbed placental weight and thickness in the GDM, activating placental insulin mediators. Exogenous insulin may enhance the expression of some fatty acid carriers in placenta by activating insulin cascade in the GDM women treated with insulin. Since maternal hyperlipemia is higher in the GDM treated with insulin, with respect to both GDMs under dietary treatment or controls, it could be desirable to reduce lipemia in these subjects to avoid excessive placental fat transfer to the fetus.

## 4. Materials and Methods

### 4.1. Ethical Approval

The study protocol was approved by the Hospital Ethics Committee (proyect 12965, GDM, Murcia, Spain) and written informed consent was obtained from all the participants.

### 4.2. Study Population

Eligible for the study were pregnant women with singleton pregnancy, 18-40 years, nonsmoking, omnivorous diet, and a fetal Doppler scan within normal reference range at recruitment. The subjects were recruited in the third trimester of gestation (28–32 weeks) in the Obstetrics and Gynecology Service of a Clinical University Hospital in Murcia, Spain Three groups of pregnant women were considered; 25 controls, 23 women with gestational diabetes mellitus receiving dietary treatment (GDM-Diet) and 20 women with gestational diabetes mellitus who required insulin treatment (GDM-Insulin).

The controls were selected among healthy pregnant women at routine echography in the 20–22 weeks of gestation. GDM was diagnosed between 24 and 28 weeks of gestation by screening with an oral challenge of 50 g glucose (O’Sullivan test) [32]. A positive screening result (1 h plasma glucose concentration >140 mg/dL) was followed by a 3-h oral glucose tolerance test with 100 g of an oral glucose load. The test was considered positive if two of the four serum glucose values were above the cut off (basal: 105 mg/dL, 1 h: 190 mg/dL, 2 h: 165 mg/dL and 3 h: 145 mg/dL) according to the criteria of the National Diabetes Data Group [19]. Metabolic control of GDM patients was assessed by the endocrinologist who decided diet or insulin based on the glycemic results on fasting glucose >90 mg/dL and 2 h postprandial glucose >120 mg/dL. Initially, all patients received a treatment based on diet and exercise. The number of calories of the diet depended on their weight gain until that moment of dietary advice, and was mainly related to carbohydrate consumption.

### 4.3. Maternal and Neonatal Anthropometrical Measurements

Fetal abdominal circumference and placental thickness were measured at both recruitment and at 38 weeks of gestation by an ultrasound scan (Voluson 730 Pro, General Electric Medical Systems, Kretz Ultrasounds, Chicago, IL, USA) the fetal biometry *z*-score was calculated using the tables of the Institute of Child Health of London [33]. Anthropometrical variables of both mother and neonate were measured at birth, and the *z*-score calculated using Spanish reference data [34].

### 4.4. Sampling

At recruitment and during labor, 10 mL of maternal blood were collected. The blood samples at recruitment were collected under fasting, but not at delivery. We also collected 2 mL of venous cord blood. Blood serum was separated within 1 h by centrifugation at 1000× *g* for 5 min. 

Samples of villous from placental central cotyledons were cut and stored at −80 °C until later analysis.

### 4.5. Biochemical Analysis

Serum glucose and triglycerides (TG) were measured using an automatic analyzer (Roche-Hitachi Modular PyD Autoanalyzer, Mannheim, Germany). Insulin was analyzed by chemiluminiscence (DIAsource INS-IRMA; Nivelles, Belgium). Insulin resistance was calculated using the homeostasis model assessment (HOMA) index.

### 4.6. Fatty Acids in Total Lipids of Serum and Placenta

Fatty acids were determined in the total lipids of maternal and cord serum, as well as in placental tissue after Folch extraction [35]. Gas chromatographic analysis was performed on a Hewlett-Packard 6890 (Agilent Technologies, Inc. Palo Alto, CA, USA) equipped with a SP-2560 capillary column (60 m × 0.25 mm, d_f_ 0.15 µm; Supelco, SIGMA-Aldrich, St. Louis, MO, USA).

### 4.7. Inhibitors and Antibodies

Inhibitors used for cell signaling studies included the PI3 kinase inhibitor LY294002 and the MEK inhibitor PD98059 (both from Sigma Aldrich, St. Louis, MO, USA) at an optimal/non cytotoxic dose of 50 µM in DMSO. Dose was selected based on previous results from dose response assays (1, 10, and 50 µM).

Primary antibodies used for Western blotting were mouse monoclonal antibodies against Fatty acid transport protein-4 (FATP-4), A-FABP, phospho-ribosomal protein S6 antibody (ser 235/236) (Santa Cruz Biotechnology, Dallas, TX, USA), FAT and LPL (Abcam, Cambridge, UK), and rabbit polyclonal antibodies to FATP-1, phospho-Akt (Ser473), phosphor-ERK (Thr 202/Tyr 204), phospho-IRS1/2 (Tyr 612) (Santa Cruz, CA, USA), anti IGF-2antibody, EL (abcam) and β-actin (Sigma Aldrich, St. Louis, MO, USA). Anti-mouse and anti-rabbit secondary antibodies conjugated with horseradish peroxidase were obtained from Santa Cruz Biotechnology.

### 4.8. Cell Culture

The human choricarcinoma-derived BeWo cell line was obtained from the European Collection of Cell Cultures (ECACC), Salisbury, UK. The cell line was authenticated by Sort Tandem Repeats (STR) profile analysis. Cells were cultured at 37 °C in F-12 medium with 10% (*v*/*v*) fetal bovine serum, 4.5 g/L glucose, 100 mg/ml streptomycin, 100 IU/penicillin, 100 IU/glutamine in a standard tissue atmosphere of 20% O_2_ and 5% CO_2_.

BeWo cells were seeded (80,000 cell per flask) and cultured for 48 h in the appropriate medium. Before the experiments, the cells were serum-starved in medium without glutamine for 3 h, and then preincubated with or without PI3 Kinase inhibitor (LY294002, 50 μM) and MEK inhibitor (PD98059, 50 μM) respectively without serum. DMSO was tested as vector control (results not shown). Preincubation was performed for 1 h with PI3K-Akt (LY294002) and MEK-ERK (PD98059) pathway inhibitors (50 μM) and stimulated with insulin (10 nmol/L) for 24 h in order to evaluate potential modifications on lipid carrier synthesis [31]. Finally protein extracts from cells were obtained for Western blotting analysis of p-AKT, A-FABP, EL, FATP-1. All the experiments were performed by triplicated.

### 4.9. Protein Extracts for Western Blotting

Protein extracts from 100 mg of placental tissue were obtained as previously detailed [36]. BeWo cells were washed twice with ice-cold PBS and then treated with cell lysis buffer (Cell Signaling Technology, Danvers, MA, USA) containing 1 mM PMSF (Sigma, Barcelona, Spain). Protein was quantified by Bradford assay [37].

### 4.10. Western Blotting Analyses

Protein extracts (30 µg from placenta and 20 µg from BeWo cells) were resolved on 12% polyacrylamide gels, and transferred onto polyvinylidene difluoride membranes (Millipore, Billerica, MA, USA). Membranes were incubated with primary antibodies overnight at 4 °C. Blots were then probed with the corresponding secondary antibody conjugated with horseradish peroxidase. Finally, membranes were stripped with Tris/HCl-Buffer pH 2.3 containing 0.1 M β-mercaptoethanol, and re-probed with anti-β-actin to perform the loading controls. Proteins were detected using a chemiluminescence Kit (Pierce ECL 2 Western blotting Substrate; Thermo Scientific, Waltham, MA, USA). Densitometry was performed on all blots using ImageJ software (National Institutes of Health, Bethesda, MD, USA). Relative protein expression data were normalized for β-actin expression.

Phosphorylated insulin signaling the mediators p-Akt, p-ERK, (Insulin receptor substrate) p-IRS-1 and p-S6 ribosomal protein, as well as the fatty acid carriers FAT/CD36, FATP-1, FATP-4, A-FABP, EL, and LPL were quantified in placenta.

### 4.11. Statistical Analysis

The results are expressed as mean ± standard error of the mean (SEM). Differences between groups were evaluated by ANOVA followed by post hoc Bonferroni analyses. For the cesarean rate, a chi2 test was performed. The minimum sample size to detect a significant difference between the groups with respect to the cord blood fatty acids (type I error α = 0.05 and type II error β = 0.2) was estimated to be 17 children/group, based on a minimal difference of 20% between means.

In the cell cultures, the differences between treatments were analyzed by student *t*-test. The significance level was set at *p* < 0.05. Pearson correlation analyses were also performed. The statistical analyses were evaluated by the SPSS^®^ 16.0 software (SPSS, Chicago, IL, USA). 

## Figures and Tables

**Table 1:** Maternal and Neonatal Anthropometric and Biochemical Features.

Mothers				
	Control (*n* = 25)	GDM-Diet (*n* = 23)	GDM-Insulin (*n* = 20)	*p*
Pregestational BMI (kg/m^2^)	23.2 ± 0.8 ^b^	26.2 ± 1 ^a,b^	28.2 ± 1.3 ^a^	0.005
Placental weight (g)	582 ± 24 ^b^	651 ± 26 ^a,b^	674 ± 34 ^a^	0.045
Placental thickness	38.2 ± 2.1 ^a^	47.8 ± 2.4 ^b^	49 ± 2.4 ^b,c^	0.002
Cesarean Rate	26%	30%	30%	0.060
Gestational age (weeks)	39.5 ± 0.15 ^a^	38.1 ± 0.3 ^b^	38.2 ± 0.2 ^b^	0.000
BMI 3rd trimester (kg/m^2^)	26 ± 0.7 ^a^	29 ± 1 ^a,b^	30.6 ± 1 ^b^	0.003
BMI at Delivery (kg/m^2^)	27.9 ± 0.7 ^b^	30.3 ± 1.0 ^a,b^	31.8 ± 1.3 ^a^	0.033
Glucose 3rd trimester (mg/dL)	72.8 ±1.4 ^a^	80.6 ±1.8 ^a,b^	84.0 ± 4.0 ^b^	0.007
Glucose delivery (mg/dL)	63.6 ± 3.7 ^a^	84.3 ± 3.7 ^b^	88.1 ± 7.4 ^b^	0.002
Insulin 3rd trimester (µIU/mL)	15.2 ± 1.4 ^a^	17.1 ± 1.7 ^a^	28.4 ± 5.0 ^b^	0.004
Insulin delivery (µIU/mL)	20.2 ± 5	20.6 ± 3.3	37.1 ± 8.1	0.067
HOMA 3rd trimester	2.7 ± 0.2 ^a^	3.4 ± 0.4 ^a,b^	5.8 ± 1.4 ^b^	0.020
HOMA delivery	3.1 ± 1.0 ^b^	4.5 ± 0.9 ^a,b^	9.9 ± 3.3 ^a^	0.039
TG 3rd trimester (mg/dL)	183 ± 17.7 ^a^	188 ± 10.6 ^a,b^	240 ± 18.3 ^b^	0.028
TG delivery (mg/dL)	222 ± 13.7	220 ± 13.5	256 ± 17.7	0.187
Total FA 3rd trimester (mg/dL)	501 ± 19.4 ^a^	506 ± 17.3 ^a^	627 ± 44.5 ^b^	0.003
Total FA delivery (mg/dL)	517 ± 21	516 ± 14.4	565 ± 18.4	0.121
**Offspring**				
z-fetal AC 3rd trimester	−0.3 ± 0.2	0.6 ± 0.2	0.6 ± 0.2	0.075
z-fetal AC delivery	−0.3 ± 0.2	0.3 ± 0.2	0.4 ± 0.2	0.071
z-Birth weight	0.3 ± 0.2	0.4 ± 0.2	0.6 ± 0.2	0.482
z-Length baby	0.2 ± 0.2	0.7 ± 0.2	0.9 ± 0.2	0.099
z-BMI baby	0.1 ± 0.25	−0.3 ± 0.2	0.02 ± 0.2	0.474
Glucose cord (mg/dL)	69.4 ± 3.7	67.8 ± 3.5	76.3 ± 6.2	0.390
Insulin cord (µIU/mL)	8.9 ± 1.7	11.3 ± 1.8	8.7 ± 1	0.453
HOMA cord	1.6 ± 0.5	1.6 ± 0.3	1.2 ± 0.2	0.706
TG cord (mg/dL)	42.1 ± 3.6 ^a^	32.1 ± 3.5 ^a,b^	28.8 ± 2.2 ^b^	0.015
Total FA cord (mg/dL)	184 ± 9.4 ^a^	146 ± 4.5 ^b^	155 ± 5.7 ^b^	0.001

Mean ± S.E.M. ANOVA was used to asses differences among the groups. Different letters (a, b and c) indicated significant differences (*p* < 0.05) between gropus. FA, Fatty acids, AC, Abdominal circumference; TG, Triglycerides; HOMA = fasting glucose (G0) (mM) × fasting insulin (I0) (μU/mL)/22.5.

## Abbreviations

GDM	Gestational Diabetes mellitus
FAT	Fatty acid translocase
FATP-1	Fatty acid transport protein
A-FABP	Fatty acid binding protein
FATP-4	Fatty acid transport protein
LPL	Lipoprotein lipase
EL	Endothelial lipase
BeWo	Human Choricarcinoma-Derived cell line
Akt	Protein Kinase B
IRS-1	Insulin receptor substrate-1
ERK	Extracellular signal regulated Kinase
S6	Ribosomal protein S6
m-TOR	Mammalian Target of Rapamycin
*z*-AC	*z*-score of ultrasound fetal abdominal circumference
